# A Comparative Study of the Phytochemical Composition, Antioxidant Properties, and In Vitro Anti-Diabetic Efficacy of Different Extracts of *Caulerpa prolifera*

**DOI:** 10.3390/md23070259

**Published:** 2025-06-21

**Authors:** Safae Ouahabi, Nour Elhouda Daoudi, Mohamed Chebaibi, Ibrahim Mssillou, Ilyesse Rahhou, Mohamed Bnouham, Belkheir Hammouti, Marie-Laure Fauconnier, Alicia Ayerdi Gotor, Larbi Rhazi, Mohammed Ramdani

**Affiliations:** 1 Faculty of Medicine and Pharmacy of Oujda, Mohammed First University, 724, Oujda 60000, Morocco; ouahabi.safae@ump.ac.ma; 2 Laboratory of Bioresources, Biotechnology, Ethnopharmacology and Health, Faculty of Sciences, Mohammed First University, BP 717, Oujda 60000, Morocco; nourelhoudada95@gmail.com (N.E.D.); mbnouham@ump.ac.ma (M.B.); 3 Higher Institute of Nursing Professions and Health Techniques, Oujda 60000, Morocco; ilyesse@hotmail.com; 4 Ministry of Health and Social Protection, Higher Institute of Nursing Professions and Health Techniques, Fez 30000, Morocco; mohamed.chebaibi@usmba.ac.ma; 5 Biomedical and Translational Research Laboratory, Faculty of Medicine and Pharmacy of Fez, Sidi Mohamed Ben Abdellah University, Fez 30000, Morocco; 6 National Agency of Medicinal and Aromatic Plants, BP 159, Principal, Taounate 34000, Morocco; mssillouibrahim@gmail.com; 7 Euromed Research Center, Euromed Polytechnic School, Euromed University of Fes (UEMF), Fes 30000, Morocco; hammoutib@gmail.com; 8 Laboratory of Chemistry of Natural Molecules, University of Liège, Gembloux Agro-Bio Tech. 2, Passage des Déportés, B-5030 Gembloux, Belgium; marie-laure.fauconnier@uliege.be; 9 Institut Polytechnique UniLaSalle, AGHYLE, UP 2018.C101, UniLaSalle, 19 rue Pierre Waguet, BP 30313, 60026 Beauvais, France; 10 Institut Polytechnique UniLaSalle, Université d’Artois, ULR 7519, 19 Rue Pierre Waguet, BP 30313, 60026 Beauvais, France; larbi.rhazi@unilasalle.fr; 11 Laboratory of Applied and Environmental Chemistry (LCAE), Faculty of Sciences, Mohammed First University, B.P. 717, Oujda 60000, Morocco; moharamdani2000@yahoo.fr

**Keywords:** *Caulerpa prolifera*, fatty acids, phenolic compounds, antioxidant activity, anti-diabetic properties, enzyme inhibition

## Abstract

The Moroccan coastline has been the focus of attention for researchers studying the national algal flora, with the aim of preserving these invaluable natural resources. Since the year 2000, these resources have stimulated great interest in the creation of new drugs, as well as their integration into food supplements and foods. Therefore, this study aims to explore the phytochemistry of a series of extracts derived from *Caulerpa prolifera*. To ensure better extraction of the various metabolites present, two extraction methods, namely maceration and the Soxhlet method, were employed using solvents of varying polarity (hexane, ethyl acetate, methanol, and water). The chemical composition of the extracts was analyzed using GC-MS for fatty acids and HPLC-DAD for phenolic compounds. Antioxidant activity was evaluated using DPPH and β-carotene bleaching assays, while antidiabetic potential was assessed by in vitro inhibition of α-amylase and α-glucosidase. In addition, Molecular docking models were employed to assess the interaction between the bioactive molecules and the human pancreatic α-amylase and α-glucosidase enzymes. Vanillin, p-coumaric acid, sinapic acid, 7,3′,4′-flavon-3-ol, and kaempferol were the most abundant phenolic compounds. Anti-diabetic and antioxidant effects were highly significant.

## 1. Introduction

Morocco, an African nation renowned for its diverse ecosystems, boasts a dual maritime frontier bordering the Western Atlantic Ocean to the west and the Mediterranean Sea to the north. This extensive coastal expanse spans 3500 km [1]. Morocco undeniably boasts a diverse range of aquatic flora and fauna. This is evidenced by the extensive study conducted by Benhissoune et al. [2], who have identified an impressive 612 species of algae in Moroccan waters [3]. The Nador Lagoon, also known as Marchica, is situated in the northern region of the country and is considered to be one of the most important Mediterranean lagoons. It boasts a diverse array of 112 algae species, including 60 rhodophyceae, 31 chlorophyceae, 19 pheophyceae, and 2 phanerogam species. Despite the richness of these algae, their potential remains largely untapped due to limited research.

*Caulerpa prolifera* (Chlorophyta) is one of the most abundant algae in the lagoon [4]. This alga is characterized by the presence of an important extracellular polysaccharide called sulfated galacotans. Wang et al. [5], have classified this alga as the fourth producer of sulfated polysaccharides. The different species of *Caulerpa* are known to occupy various environmental niches that differ considerably in terms of temperature, light availability, water movement, depth, grazing pressure, and benthic substrate [6,7,8,9,10]. The invasive potential of this genus is attributed to intrinsic characteristics that promote its adaptation to diverse habitats. Features such as phenotypic plasticity, asexual reproduction, production of surface metabolites, and nutrient absorption by rhizoids contribute to the diversity of habitats suitable for this genus [6].

The green seaweed *Caulerpa* is widely cultured, especially in ponds, because of its rapid growth rate, high antioxidant activity, and nutritional value [11,12]. *Caulerpa* has an indeterminate prostrate axis, referred to as a stolon, which attaches to a substrate by filiform rhizoids. Upright fronds are the photosynthetic and reproductive units. Fragments of the stolon can regenerate a new thallus.

*Caulerpa* species are also rich in minerals, reaching up to 55% of their dry matter [13], notably iron, calcium, magnesium, and zinc [13,14]. They contain variable levels of carbohydrates, ranging from 3.6% to 83.2% of dry matter [13]. *Caulerpa* polysaccharides are complex in composition, with a wide variety of oses (galactose, glucose, arabinose, xylose, mannose, rhamnose and fucose) [15,16,17,18], but galactose is the most important component [19], and have interesting biological properties for medical and pharmaceutical applications, such as anticoagulant, anti-inflammatory, antioxidant, anti-diabetic, immunostimulant and antitumoral effects [5]. However, the protein content in edible *Caulerpa* is relatively low compared with other protein-rich plant sources. Lipids are present in small quantities [13], but seaweeds, including *Caulerpa*, are valued for their high content of long-chain polyunsaturated fatty acids and carotenoids [20].

*Caulerpa* species are rich in chlorophylls a and b, and also contain other pigments such as siphonaxanthin [14]. In addition, they provide significant amounts of vitamins C and E (up to 46.3 and 62.7% of the recommended daily allowance (RDA), respectively, per 100 g of *Caulerpa* consumed) [13], although their nutritional value is yet to be determined. It should be noted that the *Caulerpa* genus presents a wide chemical diversity, but it is important to be cautious due to its high toxicity [21].

Recent studies have also highlighted the pro-health properties of various *Caulerpa* species, including their antioxidant and antidiabetic potential due to their richness in bioactive compounds such as polyphenols, flavonoids, and sulfated polysaccharides. These compounds are known to inhibit carbohydrate-hydrolyzing enzymes, including α-amylase and α-glucosidase, which are key targets in the management of type 2 diabetes. While species such as *Caulerpa racemosa* and *Caulerpa lentillifera* have shown promising antidiabetic activity [22,23], little is known about the antidiabetic potential of *Caulerpa prolifera*. Therefore, exploring this biological activity represents a relevant and novel aspect of its pharmacological profile.

The primary objective of this study was to conduct a comprehensive chromatographic analysis of the fatty acids and polyphenols present in *C. prolifera* extracts. Two distinct extraction methods were employed, utilizing solvents with varying polarities to facilitate a comparative assessment to optimize the recovery of bioactive compounds and better understand how the extraction method affects the biological properties of the resulting extracts. Furthermore, the research sought to evaluate the antioxidant properties of the extracts and their impact on pancreatic α-amylase and α-glucosidase activities. The outcomes of these analyses are intended to contribute to an enhanced understanding of the potential health benefits associated with *C. prolifera* extracts sourced from the Marchica lagoon, as well as their potential applications in the food and pharmaceutical sectors.

## 2. Results

### 2.1. Yields, Phenols, and Flavonoids Contents

In our recent study, we aimed to extract *C. prolifera* extracts using water, methanol, ethyl acetate, and hexane. The extraction yields ranged from 1.30% to 8.51%, and we found that the yield of extraction increases with the solvent’s increasing polarity during the extraction process. Our findings also suggest that the extraction yield of organic solvents by the Soxhlet extraction method is higher than that of the maceration extraction method.

Table 1 also shows the total phenolic content of the extracts measured using the FC method. The results of the extracts were determined based on their equivalent to gallic acid (GAE). The highest TPC value is presented by the aqueous extract at 402.34 mg GAE/g and decreases in the following order: (179.88 mg GAE/g) EAcE (M) > (112.87 mg GAE/g) EAcE (S) > (99.47 mg GAE/g) ME (M) > (53.61 mg GAE/g) ME (S).

The TFC of the extracts is also reported in Table 1. It was observed that the effect of solvents on TFC is similar to that on TPC. The highest TFC was obtained in the aqueous extract, followed by the ethyl acetate extract and the methanol extract. A similar trend was observed in the amount of TPC. This correlation between TPC and TFC assay indicates that flavonoids are the dominating phenolic group in *C. prolifera*.

### 2.2. Fatty Acid Analysis

The present study aimed to investigate the fatty acid composition of hexanic and ethyl acetate extracts of *C. prolifera* using gas chromatography (GC) and mass spectroscopy (MS). The results are presented in Table 2. The hexane extracts were predominantly composed of saturated fatty acids, with a total saturated fatty acid (TUFA) composition of 70.71% and 69.3% for HE (S) and HE (M), respectively. In contrast, the ethyl acetate extracts were mainly composed of unsaturated fatty acids, with a total unsaturated fatty acid (TUFA) composition of 58.49% and 53.73% for EAcE (S) and EAcE (M), respectively. Among the saturated fatty acids (SFA), palmitic acid was found to be the most dominant in all extracts, with a percentage of 60.21%, 59.54%, 40.76%, and 39.31% for HE (S), HE (M), EAcE (S), and EAcE (M), respectively. Among the polyunsaturated fatty acids (PUFA), linoleic acid was the most dominant in all extracts, with approximately 15.62% and 12.25% for ethyl acetate extracts and hexane extracts, respectively. Lauric acid was present in a significant amount in EAcE (~12%) while a low percentage was observed in HE (~2%). Myristic acid, 7,10-Hexadecadienoic acid, and Linolenic acid were found in relatively smaller amounts in extracts, while Eicosenoic acid and Palmitoleic acid (C16:1) were detected in minor amounts.

### 2.3. HPLC Analysis of C. prolifera Extracts

The present study aimed to investigate the chemical composition of ethyl acetate and methanol extracts of *Caulerpa prolifera* by employing HPLC-DAD analysis. The present methodology is characterized by its simplicity, ease of use, and high efficacy in the identification and quantification of major phenolic compounds in seaweed or aromatic plants. The obtained results were compared with the standards listed in Table 3, primarily based on retention time and ultraviolet spectrum.

The analysis revealed that *Caulerpa prolifera* extracts contain a diverse array of phenolic compounds, including nine phenolic acids and ten flavonoids. The extracts exhibited varying levels of abundance of the identified compounds. Sinapic acid was found to be the most abundant compound in EAcE (M) (18.54%), while Kaempferol (27%) and sinapic acid (21.28%) were the most prevalent compounds in EAcE (S). The most abundant compounds in ME (M) were 7,3,4-trihydroxy flavone (17.29%), Kaempferol (16.30%), and P-Coumaric acid (16.24%). Moreover, the extract ME (S) was found to contain a significant amount of flavonoids, primarily 7,3,4-flavon-3-ol (14.43%).

### 2.4. Antioxidant Activity

The present study reports the results of the total antioxidant activity of the green seaweed *C. prolifera* using two popular assays. The results of this study are summarized in Table 4.

The findings of this study suggest that the aqueous extract displayed the highest level of antioxidant activity, with a recorded value of 0.091 mg/mL. In comparison, the reference antioxidant, ascorbic acid, had an IC_50_ value of 0.062 mg/mL. The ethyl acetate and methanolic extracts, however, demonstrated similar levels of antioxidant activity. With a value of approximately 0.7 mg/mL, regardless of the extraction method employed. It is important to note that factors such as the chemical composition of the extracts. Algae culture conditions and extraction methods used can influence the antioxidant efficacy.

The study also showed that the ethyl acetate extracts from *C. prolifera* demonstrated the highest antioxidant activity, using the β-carotene molecule bleaching method. The recorded values for maceration and Soxhlet techniques were 0.01 mg/mL and 0.02 mg/mL, respectively. Besides, the ethyl acetate and methanol extracts manifested similar antioxidant activity, with an IC_50_ value of approximately 0.3 mg/mL. The bioactive compounds that effectively inhibited the degradation of beta-carotene were predominantly found in apolar or medium-polar extracts. Conversely, the majority of more polar extracts, including methanol and aqueous extracts, showed reduced antioxidant activity comparable to the activity of retinoic acid (RA).

### 2.5. In Vitro α-Amylase Inhibition

**Figure 1:**
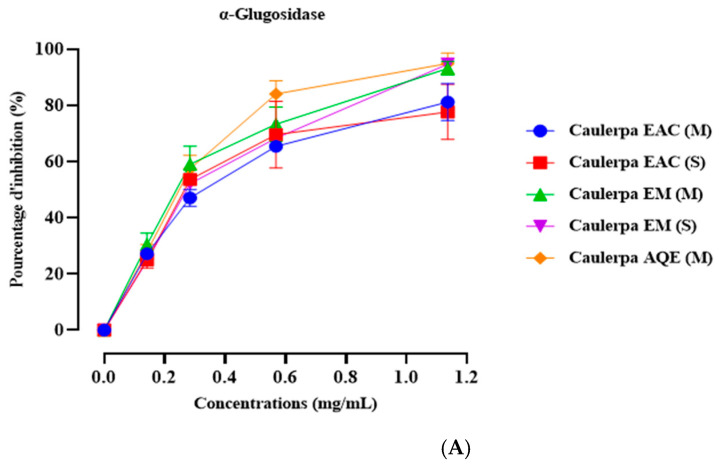

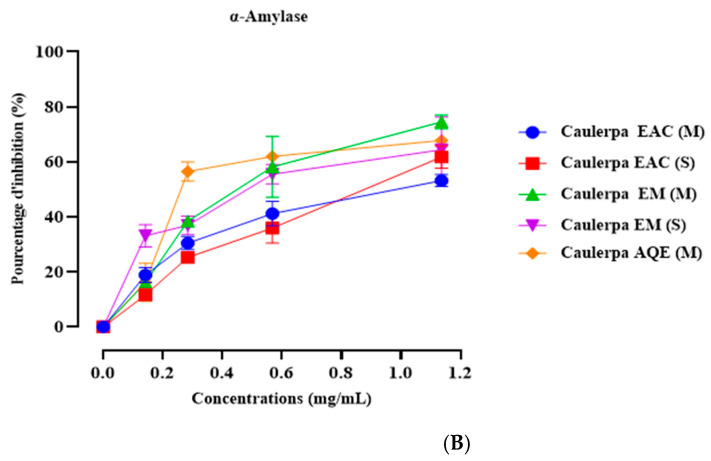
Inhibition percentage of *Caulerpa prolifera* extracts and acarbose against α-Glucosidase (**A**) and α-amylase (**B**) at different doses and their IC_50_ values. EAc: Ethyl Acetate; ME: Methanolic Extract.

Figure 1 displays the impact of *Caulerpa prolifera* extracts on α-amylase inhibitory activity, with acarbose serving as the positive control. In vitro experiments were conducted to assess the influence of different concentrations of the extracts on α-amylase enzymatic activity. The findings indicate significant inhibition of α-amylase enzymatic activity across all tested concentrations for all extracts (Table 5). Notably, the concentration of 1.14 mg/mL demonstrated the most pronounced effect, showing inhibitory activities of 53.86 ± 0.46 for EACE (M), 60.76 ± 0.99 for EACE (S), 75.20 ± 0.58 for ME (M), 64.42 ± 4.99 for ME (S), and 67.81 ± 0.34 for AQE (M) (Figure 1A). Additionally, Figure 1B presents the IC_50_ values for each extract. The results indicate that EACE (M), EACE (S) exhibit similar inhibitory effects, albeit lower than acarbose (*p* < 0.001 for both extracts, compared to acarbose). However, ME (M), ME (S), and AQE (M) demonstrate a higher inhibitory effect compared to other extracts and exhibit a statistically similar effect to acarbose.

### 2.6. Molecular Modeling Studies

The inhibition of NADPH, a crucial cofactor in various enzymatic reactions contributing to cellular antioxidant defenses, plays a vital role in regulating cellular redox balance and antioxidant defense mechanisms [24].

Concerning the chemical compounds identified by HPLC in *Caulerpa prolifera* Extracts, Rutin, 7,3′,4′-flavon-3-ol, and Quercetin were the most active molecules against NADPH oxidase with glide gscore of −6,889, −6,803, and −6,587 kcal/mol (Table 6).

**Figure 2:**
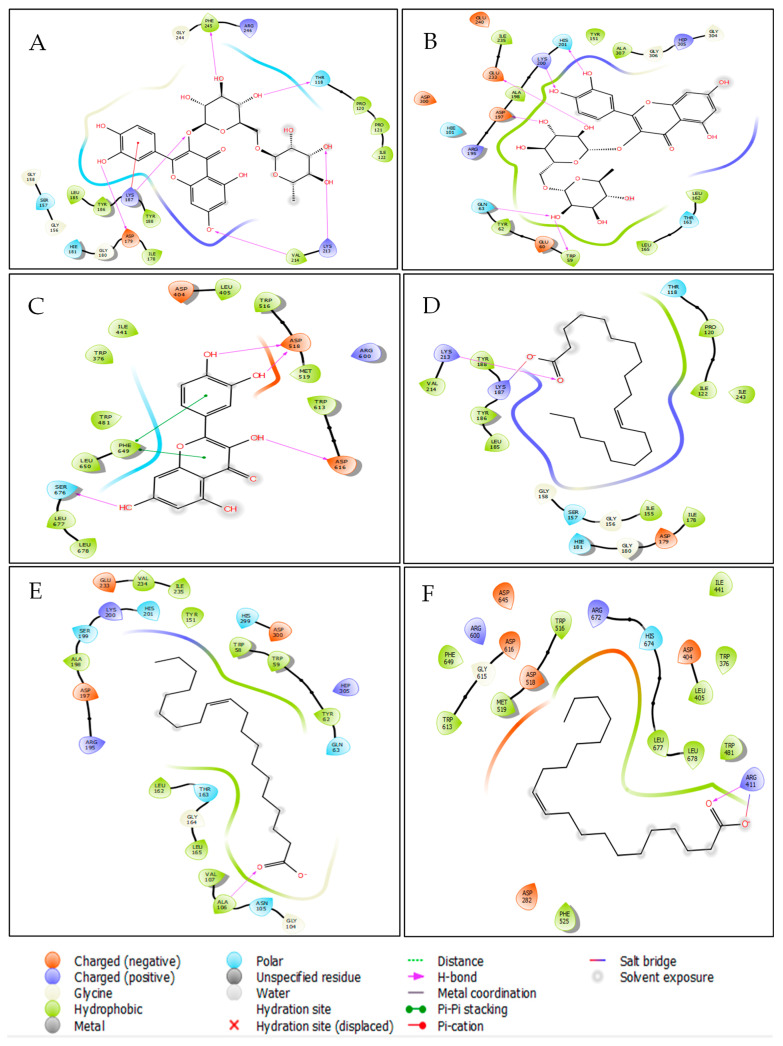
The 2D viewer of ligand interactions with the active site. (**A**,**B**): Rutin interactions with NADPHoxidase and alpha amylase active sites; (**C**): quercetin interactions with active site of alpha glucosidase; (**D**–**F**): Eicosenoic interactions with NADPHoxidase, alpha amylase, and alpha glucosidase active sites.

**Figure 3:**
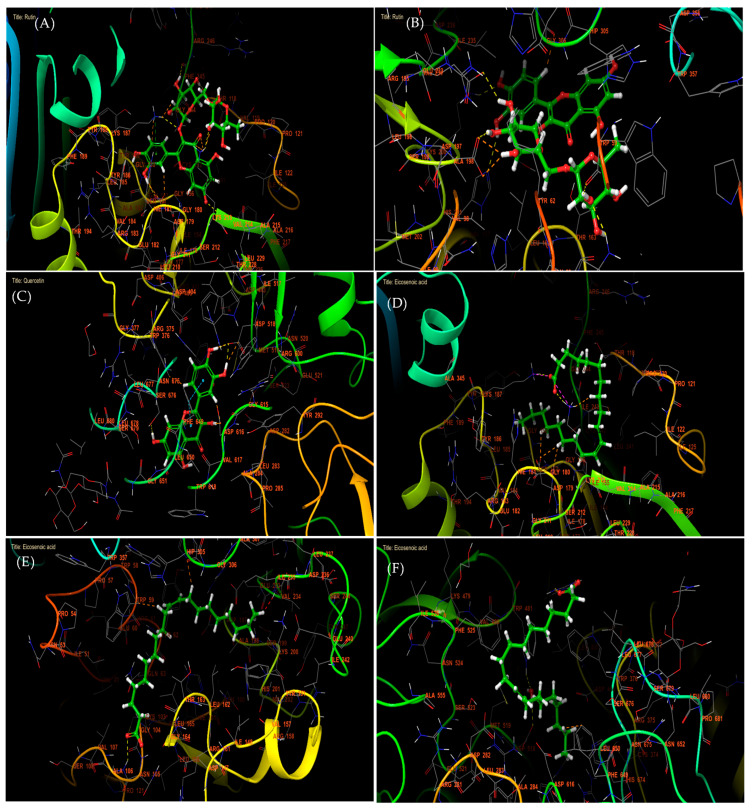
The 3D viewer of ligand interactions with the active site. (**A**,**B**): Rutin interactions with NADPHoxidase and alpha amylase active sites; (**C**): quercetin interactions with active site of alpha glucosidase; (**D**–**F**): Eicosenoic interactions with NADPHoxidase, alpha amylase, and alpha glucosidase active sites.

In antidiabetic activity, molecular docking analysis revealed that Rutin, Apigenin, and 7,3′,4′-flavon-3-ol were the most active compounds against α-amylase, with glide gscores of −7.615, −7.13, and −6.96 kcal/mol, respectively. Similarly, Quercetin, Kaempferol, and 7,3′,4′-flavon-3-ol showed the strongest binding affinity toward α-glucosidase, with glide gscores of −7.035, −5.698, and −5.558 kcal/mol (Table 6). Among the fatty acids identified in *Caulerpa prolifera* extracts, Eicosenoic acid exhibited the most notable activity against NADPH oxidase, α-amylase, and α-glucosidase, with glide scores of −3.048, −2.289, and −2.008 kcal/mol, respectively. At the molecular interaction level, Rutin formed six hydrogen bonds within the active site of NADPH oxidase (PHE 245, THR 118, LYS 213, VAL 214, LYS 187, and ASP 179) and established a π-cation bond with LYS 187 (Figure 2A and Figure 3A). Additionally, Rutin interacted with α-amylase via six hydrogen bonds (HIS 201, LYS 200, GLU 233, ASP 197, GLN 63, and TRP 59) (Figure 2B and Figure 3B). In contrast, Quercetin formed four hydrogen bonds with ASP 518, ASP 616, and SER 676 in the α-glucosidase active site, along with two π–π stacking interactions with PHE 649 (Figure 2C and Figure 3C).

Moreover, in the active site of NADPH oxidase, Eicosenoic acid established one hydrogen bond with residue LYS 213 and one salt bridge with residue LYS 187 (Figure 2D and Figure 3D). While in the active site of alpha amylase, the same molecule has established a single hydrogen bond with the residue (Figure 2E and Figure 3E). This same molecule has established one hydrogen bond and one salt bridge with residue ARG 411 (Figure 2F and Figure 3F).

## 3. Discussion

The process of obtaining phytochemicals from seaweed involves several crucial steps, including milling, grinding, homogenization, and extraction. Among these steps, extraction is considered the most vital for isolating and recovering phytochemicals from algae materials [25]. The efficiency of extraction is influenced by various factors, such as the chemical nature of phytochemicals, the extraction method used, sample particle size, the solvent chosen, and the presence of interfering substances [26,27]. The yield of extraction is dependent on the solvent’s polarity, pH, temperature, extraction time, and the composition of the sample [28]. The phytochemical profile of Caulerpa species is highly responsive to environmental conditions and growing locations. In addition, abiotic factors such as salinity, nutrient levels, temperature, and light intensity have been shown to modulate both growth and the accumulation of bioactive compounds [29]. The result of our study suggests that *Caulerpa prolifera* extracts are a rich source of diverse and potent phenolic compounds that could be utilized in various applications.

Macroalgae are considerably different from terrestrial plants in their chemical composition, physiological features, and morphology [30]. They are highly valuable sources of protein, fiber, vitamins, polyunsaturated fatty acids, macro, and trace elements, as well as essential bioactive compounds [31]. The relative composition of saturated fatty acids (SFA), monounsaturated fatty acids (MUFA), and polyunsaturated fatty acids (PUFA) is subject to variation depending on a variety of factors such as the species, location of harvest, solvent, and extraction methodology employed.

The chemical composition of EAcE and HE was determined via gas chromatography coupled with mass spectrometry (GC-MS). It highlights the presence of palmitic acid, linoleic acid, and lauric acid as major compounds. Palmitic acid is a type of fatty acid that is abundantly found in various seaweeds, including *Caulerpa racemosa* [32], *Petalonia binghamiae* (formerly *Endarachne binghamiae*) (Phaeophyceae) [33], *Asparagopsis taxiformis* (formerly *Asparagopsis sandfordiana*) [34], and *Botryocladia leptopoda* (Rhodophyta). This fatty acid is the primary type found in all three groups of seaweeds, with green seaweeds generally exhibiting the largest amount of palmitic acid, followed by red seaweeds, while brown seaweeds possess the least amount. While some similarities exist in fatty acid composition with other green seaweeds, differences are also notable. Additionally, brown and red seaweed have a higher content of myristic and stearic acids compared to green seaweed. It is noteworthy that freshwater green algae usually lack fatty acids with more than 18 carbon atoms [35,36].

High-performance liquid chromatography (HPLC) analysis identified the principal phenolic compounds present in the ethyl acetate (EAcE) and methanolic (ME) extracts. The results indicated that *C. prolifera* extracts represent a rich source of flavonoids and polyphenolic compounds. Natural medicines are widely considered to be a safer option compared to synthetic drugs, owing to their ubiquity in the human diet and wider accessibility. Furthermore, natural drugs exhibit reduced side effects and possess the potential to target multiple signaling pathways associated with tumorigenesis. Given these advantages, natural product research is burgeoning to identify new anti-cancer, antidiabetic, and antioxidant compounds not only from terrestrial plants and microorganisms but also from marine organisms. Marine organisms offer a vast assortment of pharmaceutically significant natural products, which can be employed to treat different human diseases, particularly cancer. The molecular structures of marine bioactive compounds are unique and diverse, distinguishing them from their terrestrial counterparts. Presently, there are 14 marine-based drugs available in the market, with 9 of them being employed for cancer treatment.

Numerous experimental and epidemiological studies have suggested that dietary polyphenols are essential for providing protection against various degenerative diseases. These studies, including [37,38,39], have found that polyphenols have significant health benefits, mainly attributed to their potent antioxidant properties. However, to understand their health effects fully, it is crucial to enhance our understanding of their bioavailability. Although phenolic acids constitute the primary polyphenols consumed, their bioavailability has not received as much attention as that of flavonoids.

Flavonoids, a class of polyphenols ubiquitous in plants and present in our daily diets, possess a complex molecular structure that is intricately linked to various biological functions in the human body [40]. Furthermore, flavonoids are also found in algae [41] and can be enriched by various optimization techniques. In a recent study, the presence of flavonoids and other phenolic compounds was shown to confer antioxidant protection against lipid peroxidation in rat liver microsomes exposed to the oxidizing agent carbon tetrachloride [42,43]. Notably, the results of this investigation were comparable to those observed with the commercial antioxidants BHT and BHA.

The present study also reports the total antioxidant activity of the green seaweed *C. prolifera* using two popular assays. Namely, the DPPH free radical scavenging assay and the β-carotene bleaching assay. The study employed several solvents, including water, methanol, and ethyl acetate, to extract the seaweed’s bioactive compounds. It is worth mentioning that the choice of solvents plays a vital role in extracting various chemical substances from the plant material, which consequently affects the antioxidant capacity. These findings are consistent with previous research [44]. The findings indicated that the aqueous extract, characterized by a high total phenolic content, exhibited strong antioxidant activity. These results provide valuable insights into the potential utilization of *C. prolifera* extracts as functional ingredients in the food and pharmaceutical industries.

The in vitro anti-diabetic activities of *C. prolifera* extracts were investigated through their potential to inhibit pancreatic enzyme activity. The inhibition of alpha-amylase and alpha-glucosidase is a key mechanism in the anti-diabetic activity [45]. Alpha-amylase is an enzyme found in saliva and the pancreas that breaks down polysaccharides (such as starch) into simple sugars. Inhibiting this enzyme slows down the breakdown of complex carbohydrates into simple sugars, thereby reducing the rise in blood glucose levels after meals. Therefore, alpha-amylase inhibitors can help control blood sugar levels in individuals with diabetes by delaying carbohydrate absorption.

Alpha-glucosidase is an enzyme located in the intestinal wall that breaks down complex sugars into simple sugars, facilitating their absorption into the bloodstream. Inhibiting this enzyme delays carbohydrate absorption and also helps reduce postprandial blood sugar spikes.

Diabetes mellitus is a metabolic endocrine disorder characterized by disruptions in the metabolism of carbohydrates, leading to persistent high levels of blood sugar [46]. This increase in blood glucose is a result of insufficient insulin secretion and/or inadequate responsiveness to this hormone [47]. Uncontrolled chronic hyperglycemia is linked to complications, notably impacting the eyes, kidneys, heart, nerves, and blood vessels [48]. Furthermore, it is established that oxidative stress plays a role in the onset of diabetes and the progression of its complications, particularly through the generation of free radicals [49,50]. Hence, the need for diabetes control. Several scientific studies have experimentally demonstrated the interest of natural resources in improving the metabolic dysregulation induced by diabetes mellitus, contributing notably to the maintenance of glycemic homeostasis [51]. Therefore, the aim of our work was to evaluate the effect of one of the marine green algae on diabetes. To achieve this, we studied the impact of *Caulerpa prolifera* on digestive enzymes related to sugar catalysis, using in vitro experiments.

The main outcomes of the in vitro investigation regarding the inhibitory impact of α-amylase and α-glucosidase showed that each *Caulerpa prolifera* tested extract demonstrated notable inhibition of α-amylase enzymatic activity across all tested concentrations. Besides, IC_50_ values indicated comparable inhibitory effects among EACE (M) and EACE (S), all exhibiting lower activity compared to acarbose, a potent α-amylase and α-glucosidase inhibitor [52]. However, ME (M), ME (S), and AQE (M) exhibited higher inhibitory activity than the other extracts, showing a statistically similar effect to acarbose. This suggests that the extraction method of this algae has no influence on enzyme inhibition, as results remain consistent regardless of whether the extraction method used is Soxhlet or maceration. However, the type of extract (Ethyl Acetate, Methanolic Extract, or Aqueous Extract) significantly influences the results, indicating varied inhibitory effects. Our findings align with our previous research, indicating that the methanolic extract and aqueous extract obtained from maceration and Soxhlet possess the same effect and a potent effect than the Ethyl Acetate extract [53].

This consistency underscores the robustness of our results across different extraction methods and reinforces the notion that the choice of solvent significantly influences the inhibitory activity of the extracts. The biological activity of the identified compounds from *Caulerpa prolifera,* particularly their inhibitory effects on α-amylase and α-glucosidase, can be linked to their molecular structures. The results could be related to the presence of phenolic compounds, flavonoids, and tannins, with high concentrations in the methanolic extract [54,55]. According to [56], the methanolic extract of *Caulerpa prolifera* includes polyphenols in an amount of 66.61 ± 1.14 mg GAE/g DE. Furthermore, it includes tannins (19.06 ± 6.50 mg CE/g DE) and a higher flavonoid content (114.16 ± 0.91 mg QE/g DE). However, the specific phenolic compounds present in the methanolic extract of *Caulerpa prolifera* have not been discussed yet. According to [57] *Caleurpa* spp. have various flavonoids, such as kaempferol and quercetin. These flavonoids have been correlated with antioxidant activity, which may explain the beneficial effects of the extract on diabetes by its ability to inhibit α-amylase and α-glucosidase. These enzymes are involved in carbohydrate digestion, and their inhibition can help regulate blood glucose levels, which is beneficial for individuals with diabetes [58]. Additionally, *Caulerpa prolifera* contains fatty acids such as Tridecanoic acid, Tetradecanoic acid, hexadecanoic acid, cis-10-heptadecenoic acid, and sterols, including Stigmast-5-en-3-ol, (3.beta.)-, Stigmast-5-en-3-ol, oleate, Carvacrol, Phytol, Phytol acetate, and Squalene (all E) [56]. These compounds may also play a crucial role in anti-α-amylase and anti-α-glucosidase effects [59,60,61].

## 4. Materials and Methods

### 4.1. Chemicals and Reagents

Analytical-grade chemicals and reagents were procured from Merck (Darmstadt, Germany) and Carl Roth GmbH (Karlsruhe, Germany) to determine the total phenolic and flavonoid components. Methanol, ethyl acetate, and N-hexane were acquired from Merck (Darmstadt, Germany). α-amylase, α-glucosidase, β-carotene, 1,1-Diphenyl-2-picrylhydrazyl (DPPH•), acarbose, and 3,5-Dinitrosalicylic acid (DNSA) were obtained from Merck (Sigma-Aldrich, St. Louis, MO, USA). Phenolic standards, including ascorbic acid, kojic acid, gallic acid, apigenin, succinic acid, cholesterol, and tannic acid, were sourced from Merck and Carl Roth GmbH (Karlsruhe, Germany).

### 4.2. Plant Material and Extraction

In April 2021, the green algae species *C. prolifera* were harvested from the Nador lagoon located at 35°08′26.9″ N 2°29′09.6″ W in northern Morocco.

**Figure 4:**
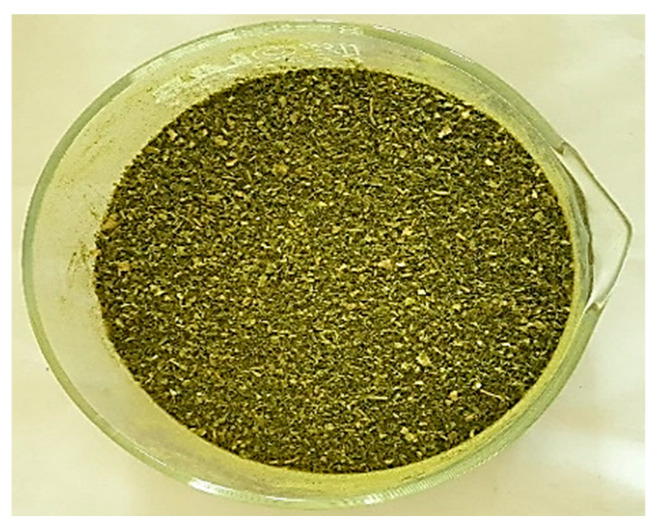
Powder of dried *C. prolifera*.

The collected seaweed sample was transported to the laboratory for further processing. *C. prolifera* was carefully cleaned and rinsed with distilled water before being exposed to light for 48 h. The dried sample was then placed in an oven at 35 °C for 24 h, as illustrated in Figure 4. The seaweed was then lyophilized and ground into a fine powder for extraction purposes. To extract the valuable components, we utilized maceration and Soxhlet techniques with four solvents: hexane, ethyl acetate, methanol, and distilled water [62,63,64]. The resulting extracts were then filtered using a glass filter crucible (50 mL, with Porosity 4, Isolab, Wertheim, Germany) and placed in flasks. The extracts were then dehydrated using a rotary evaporator (Laborota 4000, Heidolph Instruments, Schwabach, Germany). This thorough extraction process ensures that the resulting extracts are of the highest quality, making them ideal for further studies, and the extraction yield was calculated using the following Equation (1):

(1) $$Yield(\%)=\frac{mass\;dried\;extract\;(g)}{mass\;dried\;matrix\;(g)}\times 100$$

The recorded data is the median of three extraction replicates.

#### 4.2.1. Maceration Extraction

To extract soluble compounds from a solid substance, maceration is the most common method, involving immersion in a cold liquid. In this case, extracts were prepared by stirring 100 g of macroalgae powder with 200 mL of solvent of increasing polarity (99% n-hexane for 2 h, ethyl acetate for 24 h, methanol for 24 h, and distilled water for 24 h) at room temperature.

#### 4.2.2. Soxhlet Extraction

In order to extract active compounds from marine macroalgae, we utilized the alternative technique, namely the Soxhlet extraction method. This method involves employing a Soxhlet chamber, an extraction flask, and a condenser [47]. The process begins by placing 35 g of marine macroalgae powder into an extraction thimble, which is then combined with 300 mL of a selected solvent (such as hexane, ethyl acetate, or methanol) in the extraction flask. The duration of the Soxhlet extraction process is determined by the time it takes to extract all the soluble compounds with affinity for the solvent at a given temperature.

### 4.3. Phytochemical Compounds

#### 4.3.1. Quantification of Total Phenolic Constituents

The study aimed to determine the total amount of polyphenols present in *C. prolifera* extracts. To achieve this, a modified Folin–Ciocalteu method was used. In this method, 200 µL of the extract solution with a concentration of 1 mg/mL was mixed with 1000 µL of Folin–Ciocalteu reagent dissolved in distilled water, followed by the addition of 800 µL of sodium carbonate (75 g/L). The resulting mixture was then incubated at room temperature for one hour in the dark. After incubation, absorbance readings were taken at 765 nm using a spectrophotometer, with ethanol serving as a control. To generate calibration curves, the concentration of gallic acid was altered (ranging from 0 to 0.1 mg/mL). All experiments were conducted in triplicate to obtain mean values and their corresponding standard deviations. The total amount of phenolic compounds was expressed in milligrams of gallic acid equivalents per gram of dry extract (mg GAE/g) [53].

#### 4.3.2. Measurement of Total Flavonoid Content

200 µL of each *C. prolifera* extract, 1000 µL of distilled water, and 50 µL of NaNO_2_ (5%) were combined. Subsequently, after a 6-min incubation period, 120 µL of AlCl3 (10%) was introduced, followed by a further 5-min incubation period at room temperature in darkness. This was followed by the addition of 400 µL of 1 M NaOH and 230 µL of distilled water. The absorbance was then measured at 510 nm. To construct the calibration curve, various concentrations of quercetin solution (ranging from 0 to 0.1 mg/mL) were utilized as standards. Each measurement was conducted in triplicate to ensure result reproducibility [65].

### 4.4. Fatty Acid GC-MS Analysis of C. prolifera Extracts

In line with the methodology outlined in Loukili et al. [66], methyl esters of hexane fatty acids and ethyl acetate were extracted from *C. prolifera* with certain adaptations. The Shimadzu GC system (Kyoto, Japan) equipped with a BPX25 capillary column featuring a 5% diphenyl and 95% dimethylpolysiloxane phase (30 m × 0.25 mm × 0.25 µm) (Kyoto, Japan) coupled with a QP2010 MS was utilized for the characterization and identification of fatty acids. Helium gas (99.99%) served as the mobile phase with a flow rate set at 3 L/min. The temperature settings for the injection, ion source, and interface were maintained at 250 °C. The column oven temperature program began at 50 °C for 1 min, followed by a gradual increase to 250 °C at a rate of 10 °C/min, and held for 1 min. Sample components underwent ionization in Electron Ionization (EI) mode at 70 eV, scanning a mass range of 40 to 300 m/z. Each extract, in a volume of 1 µL, was injected in split mode, and triplicate analyses were conducted for each sample. Compound characterization relied on comparisons of retention times, mass spectra fragmentation patterns, and databases, including the National Institute of Standards and Technology’s database (NIST). Data processing was conducted using LabSolutions (version 2.5, Shimadzu, Kyoto, Japan).

### 4.5. HPLC Analyses of C. prolifera Extracts

Identification of phenolic compounds within the ethyl acetate and methanolic extracts was accomplished utilizing High-Performance Liquid Chromatography (HPLC) employing an Agilent 1100 system (Agilent Technologies, Palo Alto, CA, USA) coupled with a diode array UV detector (Bruker, Berlin, Germany). Each extract (10 µL) underwent separation through a Zorbax XDB-C18 column (5 µm, 250 × 4.6 mm) connected to the Agilent 1100 system, preceded by a 4 × 3 mm C18 cartridge precolumn (Agilent Technologies). The elution gradient protocol employed was as follows: 0 to 5 min with 95% solvent A and 5% solvent B, 25 to 30 min with 65% solvent A and 35% solvent B, 35 to 40 min with 30% solvent A and 70% solvent B, and finally 40 to 45 min with 95% solvent A and 5% solvent B. Solvent A comprised water/methanol (9/1) with 0.1% phosphoric acid, while solvent B consisted of methanol with 0.1% phosphoric acid. The elution was carried out at a constant flow rate of 1 mL/min under a temperature of 40 °C. Spectrophotometric data were collected at wavelengths of 254 nm, 280 nm, 320 nm, 370 nm, and 510 nm. Compound identification was conducted by comparing their retention times and UV spectra with those of standard compounds.

### 4.6. Antioxidant Activity

#### 4.6.1. Scavenging 2,2-Diphenyl-1-picrylhydrazyl Radical Test

The antioxidant activity of different extracts of *C. prolifera* was assessed using the 1,1-diphenyl-2-picrylhydrazyl (DPPH) radical bleaching method, following the protocol outlined by Brand-Williams et al., [67] with minor adjustments. The initial concentration of both the extracts and Ascorbic acid was maintained at 1 mg/mL. A solution comprising 0.8 mL of samples or standard (ascorbic acid) at varying concentrations (ranging from 0.02 to 0.32 mg/mL) and 2 mL of DPPH• solution (prepared by dissolving 2 mg of DPPH• in 200 mL of MeOH) was prepared and manually agitated. Following a 30-min incubation period in darkness at room temperature, the absorbance of the samples was measured using a UV/visible spectrophotometer at a wavelength of 517 nm, relative to the blank. Each analysis was conducted in triplicate.

The inhibitory activity of the DPPH radical by a sample was determined using the following Equation (2):

(2) $$Inhibition\;Percent=\left[\frac{\left(Ab-As\right)}{Ab}\right]\times 100$$

where *Ab*: Absorbance of the blank, *As*: Absorbance of a sample (or positive control).

The graph plotting inhibition percentage against extract concentration was used to calculate the IC_50_.

#### 4.6.2. β-Carotene Bleaching Assay

The antioxidant potential of *C. prolifera* extracts was assessed using the method described by Ref. [68]. This evaluation relied on the extracts’ capacity to mitigate oxidative damage to β-carotene in an emulsion, employing the carotene bleaching assay. To prepare the emulsion, 2 mg of β-carotene was dissolved in 10 mL of chloroform, supplemented with 20 mg of linoleic acid and 200 mg of Tween 80 as an emulsifier. The chloroform was evaporated under vacuum at 40 °C, followed by the addition of 100 mL of distilled water while vigorously stirring the solution. Subsequently, 0.2 mL of the emulsion was dispensed into individual test tubes, along with either the extract or a reference antioxidant solution (BHA) at a concentration of 1 mg/mL. The absorbance was recorded at 470 nm using a 96-well microplate reader at t0 (immediately after emulsion addition) and after a 2-h incubation period.

The inhibition of the linoleate/β-carotene radical was determined using the following Equation (3):

(3) $$\mathrm{Bleaching}\;\mathrm{inhibition}\;(\%)=100-\frac{initial\;\left(\beta -carotene\right)\left(t0\right)-\left(\beta -carotene\right)\;after\;2h\;}{initial\;\left(\beta -carotene\right)\left(t0\right)}\times 100$$

### 4.7. In Vitro α-Amylase Inhibition

The inhibitory activity of various extracts of *C. prolifera* against α-amylase was assessed following the protocol outlined by Daoudi et al. [48]. A reaction mixture was prepared, comprising 0.2 mL of the sample or acarbose (utilized as a positive control at concentrations ranging from 2.27 to 0.14 mg/mL), 0.2 mL of phosphate buffer (pH 6.9), and 0.2 mL of enzyme solution (13 IU). This mixture was pre-incubated at 37 °C for 10 min before the addition of 0.2 mL of enzyme-substrate solution (1% starch dissolved in phosphate buffer). The reaction proceeded at 37 °C for 30 min. The enzymatic reaction was halted by adding 0.6 mL of DNSA reagent, followed by an incubation step at 100 °C for 8 min, followed by cooling in a cold-water bath. Absorbance was measured at 540 nm.

The inhibition percentage was calculated using the following Equation (4):

(4) $$Inhibition\;activity\left(\%\right)=\frac{\left(OD\;control\;540\;nm-OD\;control\;blank\;540\;nm\right)-(OD\;sample\;540\;nm-OD\;sample\;blank\;540\;nm)}{OD\;control\;540\;nm-OD\;control\;blank\;540\;nm}\times 10$$

The IC_50_ of the various test was done graphically using the function:

(5) $$Inhibition\;percentage\left(\%\right)=f(\log\left(sample\;concentration\right))$$

### 4.8. In Vitro α-Glucosidase Inhibition Assay

The effect of *C. prolifera* extracts on intestinal α-glucosidase activity was evaluated using a modified version of the protocol outlined by Hbika et al. [69]. A mixture was prepared by combining 100 mL of sucrose (50 mM), 1000 mL of phosphate buffer (50 mM, pH 7.5), and 100 mL of intestinal α-glucosidase enzyme solution (10 IU). This mixture was then supplemented with 10 mL of distilled water (as a control), acarbose (as the positive control), or *C. prolifera* extract solution at a concentration of 2.2 g/mL. Subsequently, the mixture was incubated for 25 min at 37 °C in a water bath. To terminate the enzymatic reaction and quantify the release of glucose, the mixture was heated at 100 °C for 5 min:

(6) $$Inhibition\;activity\left(\%\right)=\frac{\left(OD\;control\;540\;nm-OD\;control\;blank\;540\;nm\right)-(OD\;sample\;540\;nm-OD\;sample\;blank\;540\;nm)}{OD\;control\;540\;nm-OD\;control\;blank\;540\;nm}\times 10$$

### 4.9. Theoretical Study

#### 4.9.1. Ligands Preparation

To evaluate the antioxidant and antidiabetic activities of the compounds identified in *Caulerpa prolifera* extracts, a comprehensive molecular analysis was performed. All chemical compounds identified by HPLC Analysis or by GC/MS in *Caulerpa prolifera* extracts were downloaded from the PUBCHEM platform in .SDF format. Then, these molecules were prepared using the LigPrep subsystem in Maestro 11.5 from the Schrödinger suite (version 2018, Schrödinger). The minimization process employed the OPLS3 force field, and the generation of all possible ionic states was achieved at a target pH of 7.2  ±  2 using Epik. Additionally, for each ligand, plausible stereo isomers and lower-energy ring conformations were generated [70].

#### 4.9.2. Receptor Preparation

The structure of human NADPH oxidase (PDB ID: 2CDU) [71], and the alpha amylase (PDB ID: 1B2Y) [38] and alpha glucosidase (PDB ID: 5NN8) [53] were downloaded from the PDB data bank. The protein preparation wizard of the Schrödinger suite was utilized to process the receptor, involving tasks such as assigning bond orders, adding hydrogen, filling empty side chains and loops with PRIME, and ultimately removing all water from the crystal structures. Following the optimization of these crystal structures, a restrained minimization with a root mean square deviation (RMSD) of 0.3 Å was performed using the OPLS3 force field [72].

#### 4.9.3. Grid Generation and Molecular Docking

The minimized protein structures were then utilized to generate grids through the “Receptor Grid Generation” panel. For each protein, a grid was established with default parameters, including a Van Der Waals scaling factor of 1.00 and a charging cut-off value of 0.25, in accordance with the OPLS3 force field. A cubic receptor grid box with dimensions of 20 Å × 20 Å × 20 Å was centered on the selected co-crystallized ligand. The molecular docking assay utilized the Standard Precision (SP) scoring method of Glide, integrated into the Schrödinger suite-Maestro version 12.5 [73].

## 5. Conclusions

Macroalgae are a highly promising resource for the future due to their high nutritional value and versatile applications in various fields such as food, energy, medicine, cosmetics, and biotechnology. To explore the potential of macroalgae, a study was conducted to analyze seven extracts of *Caulerpa prolifera* and to determine their nutritional properties. The results revealed that the methanolic and ethyl acetate extracts contained a significant amount of Sinapic acid, Kaempferol, 7,3,4-trihydroxy flavone, and P-Coumaric acid. The hexanic and ethyl acetate extracts were rich in Palmitic acid and linoleic acid, which are the main fatty acids detected. The ethyl acetate extracts demonstrated strong antioxidant activity by the beta-carotene bleaching method, while the aqueous extract of *C. prolifera* exhibited remarkable antioxidant properties against the DPPH method. The aqueous extract also demonstrated a strong capacity to inhibit pancreatic α-amylase and intestinal α-glucosidase enzymes involved in sugar degradation, which can be attributed to the high content of polyphenols and flavonoids. The findings of this study hold significant importance in the development of commercial products based on cultivated marine macroalgae and can provide a novel solution for the sustainable production of biomass.

## Figures and Tables

**Table 1:** Phenols and flavonoids contents of different extracts of *C. prolifera*.

Solvent	Extraction Methods	Yield (%)	Polyphenols (mg GAE/g)	Flavonoids (mg QE/g)
Hexane	M	1.30 ± 0.01	-	-
	S	2.23 ± 0.02	-	-
Ethyl acetate	M	4.46 ± 0.03	179.88 ± 0.03	154.64 ± 0.02
	S	6.81 ± 0.05	112.87 ± 0.07	40.35 ± 0.04
Methanol	M	7.12 ± 0.02	99.47 ± 0.06	22.93 ± 0.01
	S	8.41 ± 0.04	53.61 ± 0.02	16.70 ± 0.03
Water	M	8.51 ± 0.02	402.34 ± 0.08	196.65 ± 0.09

GAE: Gallic Acid Equivalent; QE: Quercetin Equivalent; M: Maceration; S: Soxhlet.

**Table 2:** Fatty acid composition of hexane and ethyl acetate extracts from green algae *C. prolifera*.

Fatty Acids	RT (min)	HE (%)		EAcE (%)	
		M	S	M	S
Lauric acid (C1:0)	17.83	2.61 ± 0.03	2.31 ± 0.02	12.13 ± 0.04	12.45 ± 0.05
Eicosenoic acid (C20:1)	20.08	3.58 ± 0.02	3.20 ± 0.02	5.52 ± 0.03	nd
Myristic acid (C14:0)	20.39	7.15 ± 0.04	8.19 ± 0.04	2.29 ± 0.01	5.28 ± 0.02
7,10-Hexadecadienoic acid (C16:2)	21.17	7.09 ± 0.02	6.52 ± 0.03	9.32 ± 0.03	9.50 ± 0.04
Palmitoleic acid (C16:1)	23.11	1.19 ± 0.01	2.15 ± 0.02	6.35 ± 0.02	6.81 ± 0.03
Palmitic acid (C16:0)	23.31	59.54 ± 0.06	60.21 ± 0.05	39.31 ± 0.12	40.76 ± 0.08
Linoleic acid (C18:2)	25.03	12.25 ± 0.03	10.14 ± 0.03	15.62 ± 0.05	15.66 ± 0.04
Linolenic acid (C18:3)	25.09	6.59 ± 0.02	7.28 ± 0.03	9.46 ± 0.03	9.49 ± 0.03
SFA ^a^		69.30	70.71	53.73	58.49
UFA ^b^		30.70	29.29	46.27	41.46
UFA/SFA ^c^		0.44	0.41	0.86	0.71

RT: Retention time; M: maceration; S: Soxhlet, HE: Hexane Extract; EAcE: Ethyl acetate Extract; nd: not detected; ^a^: saturated fatty acids (SFA); ^b^: unsaturated fatty acids (UFA); ^c^: unsaturation ratio = UFA/SFA.

**Table 3:** Chemical composition of ethyl acetate and methanolic extracts from green algae *C. prolifera*.

N°	Compounds	RT (min)	EAcE (%)		ME (%)	
			M	S	M	S
1	Gallic acid	15.47	nd	0.99	nd	0.36
2	Catechin	18.68	1.23	0.87	1.94	0.90
3	4-hydroxy-benzoic acid	18.91	0.39	1.75	nd	0.87
4	Chlorogenic acid	19.15	0.63	1.50	2.55	0.77
5	Caffeic acid	19.45	0.47	2.22	0.91	0.74
6	Syringic acid	19.74	1.36	1.72	1.99	1.01
7	Vanillin	23.10	13.16	2.52	10.48	8.94
8	p-Coumaric acid	23.63	3.72	nd	16.24	12.84
9	Sinapic acid	24.09	18.54	21.28	7.27	6.82
10	7,3′,4′-flavon-3-ol	24.92	12.26	7.77	17.29	14.43
11	Rutin	25.16	3.27	6.03	nd	7.07
12	Salicylic acid	25.32	2.27	4.99	3.21	4.38
13	Quercetin	25.46	3.98	3.13	2.72	3.30
14	Cinnamic acid	25.48	5.98	6.05	6.85	11.13
15	Luteolin	25.64	2.86	3.52	1.67	nd
16	Apigenin	25.87	4.59	4.90	4.59	9.54
17	Kaempferol	26.10	6.03	27.00	16.30	2.99
18	Flavone	26.92	3.71	nd	nd	3.07
19	Flavonone	27.41	15.57	3.75	6.00	10.85

RT: Retention Time; M: maceration; S: Soxhlet; EAcE: Ethyl acetate Extract; ME: Methanolic Extract; nd: not detected.

**Table 4:** IC_50_ values of *C. prolifera* extracts.

Extracts		IC_50_ (mg/mL)	
		DPPH	β-Carotene
EAcE	M	0.702 ± 0.311	0.01 ± 0.18
	S	0.767 ± 0.063	0.02 ± 0.25
ME	M	0.691 ± 0.041	0.29 ± 0.09
	S	0.723 ± 0.020	0.31 ± 0.31
AQE	M	0.091 ± 0.091	0.381 ± 0.11
Ascorbic Acid		0.06	-
BHA		-	0.02

EAcE: Ethyl Acetate extract; ME: Methanolic extract; AQE: Aqueous extract; M: Maceration; S: Soxhlet.

**Table 5:** IC_50_ values of *C. prolifera* extracts and acarbose in α-amylase and α-glucosidase inhibition.

Inhibitors	IC_50_ (mg/mL)		
	α-Amylase		α-Glucosidase
**Acarbose**		0.35 ± 0.08	0.39 ± 0.04
**EAcE**	M	0.88 ± 0.08	0.48 ± 0.02
	S	0.83 ± 0.01	0.43 ± 0.07
**ME**	M	0.62 ± 0.11	0.35 ± 0.08
	S	0.63 ± 0.14	0.29 ± 0.05

EAcE: Ethyl Acetate extract; ME: Methanolic extract; AQE: Aqueous extract; M: Maceration; S: Soxhlet.

**Table 6:** Docking results in ligands in different receptors.

Compound Name		Glide Gscore (Kcal/mol)		
		NADPH Oxidase (PDB: 2CDU)	Alpha Amylase (PDB: 1B2Y)	Alpha Glucosidase (PDB: 5NN8)
Chemical compounds by HPLC analysis	4-hydroxybenzoic acid	−5.355	−5.016	−4.366
	7,3,4-flavon-3-ol	−6.803	−6.961	−5.558
	Apigenin	−6.405	−7.130	−5.202
	Caffeic acid	−5.484	−5.953	−4.237
	Catechin	−5.550	−6.283	−4.908
	Chlorogenic acid	−4.812	−6.254	−3.738
	Cinnamic acid	−4.637	−3.713	−3.353
	Flavone	−5.040	−5.175	−4.326
	Flavonone	−5.192	−5.389	−4.624
	Gallic acid	−5.878	−5.333	−4.32
	Kaempferol	−5.543	−6.617	−5.698
	Luteolin	−6.574	−6.807	−5.425
	p-Coumaric acid	−5.017	−5.742	−3.558
	Quercetin	−6.587	−6.817	−7.035
	Rutin	−6.889	−7.615	−5.237
	Salicylic acid	−5.469	−4.565	−3.911
	Sinapic acid	−5.299	−4.35	−3.543
	Syringic acid	−6.132	−5.973	−3.472
	Vanillin	−6.603	−6.012	−4.651
Fatty Acid by GC-MS	7,10-Hexadecadienoic acid	−1.001	-	-
	Eicosenoic acid	−3.048	−2.289	−2.008
	Lauric acid	-	-	-
	Linoleic acid	−0.817	-	-
	Linolenic acid	−0.546	-	−0.873
	Margaric acid	-	-	-
	Myristic acid	-	-	-
	Oleic acid	−0.665	-	-
	Palmitic acid	−0.006	-	-
	Palmitoleic acid	-	-	-
	Stearic acid	−0.552	-	-

## Data Availability

Data are available upon request to the authors.
